# Association between age, gender and multimorbidity level and receiving home health care: a population-based Swedish study

**DOI:** 10.1186/s13104-015-1699-2

**Published:** 2015-11-24

**Authors:** Andrzej Zielinski, Anders Halling

**Affiliations:** Lyckeby Primary Healthcare Centre and Blekinge Centre of Competence, Källevägen 12, 371 62 Lyckeby, Sweden; Research Unit of General Practice, Institute of Public Health, University of Southern Denmark, J.B. Winsløws Vej 9a, 5000 Odense, Denmark

**Keywords:** Primary care, Geriatric medicine, Public health, Multimorbidity

## Abstract

**Background:**

Home health care is an important part of primary health care. How delivery of home health care is organised is probably important for sustainability of the healthcare system as a whole. More than 50 % of individuals over 65 years old have multimorbidity, which increases with higher age, also influencing the needs of home health care. Our aim was to study the proportion of the population above 65 years receiving home health care according to age, gender and multimorbidity level.

**Methods:**

The study population comprised 32,130 people aged 65 or more, living in Blekinge County in southern Sweden. We analysed data from patient electronic medical records for patients receiving home health care delivered in patients’ own homes by nurses, physiotherapists and occupational therapists. We used the Adjusted Clinical Groups Case-Mix System in order to group individuals according to diagnoses into six levels of multimorbidity. In order to analyse the differences between individuals receiving home health care and those who did not, we used Chi squared test. Logistic regression analysis was conducted in order to study how the dependent variable was influenced by the independent variables.

**Results:**

A total of 7860 (28 %) of the studied population received home health care in 2011. Logistic regression analysis showed that men had 26 % lower odds of receiving home care compared to women (OR = 0.74, 95 % CI 0.69–0.78). There was also a substantial group (22 %) with low multimorbidity level among people receiving home health care. Adjusting for gender and age showed no differences in odds of receiving home health care for patients with lower levels of multimorbidity. However, for patients with higher levels of morbidity the odds increased dramatically for both genders.

**Conclusion:**

The question of to whom and to what extent home health care should be provided is an important challenge for policy makers. Our results show that there are differences in the use of home health care dependent on gender, age and multimorbidity level, but also that home health care is provided to individuals with low morbidity. Further studies could explain the factors influencing home health care use.

## Background

In Western Europe the proportion of the population in the labour market is decreasing due to the increase in the number of retired individuals. The number of elderly people with more than one disease is increasing [1]. Home health care is an important part of primary healthcare (PHC) in most Western European countries and provided to the patients in their own homes after needs assessment. In Sweden, home health care can be provided by different stakeholders (councils, municipalities, private), but is an important part of PHC. It is essential so that patients can live an independent life as long as possible in their own homes, and so that they are discharged from hospital as soon as medically possible. This is a holistic assessment of the patient’s possibility of living with his/her disease(s) in his/her life situation (family, housing, economy etc.). Home health care represents a large economic challenge to society, and earlier studies have shown that costs of home health care are high [2]. How delivery of home care is organised is probably important for sustainability of the healthcare system, as home health care is complex and can involve many different professionals [3]. It has previously been shown that home health care influences patient satisfaction and quality of care for high-risk patients with chronic diseases [4]. Home health care also decreases number of GP consultations [5].

More than 50 % of individuals over the age of 65 have multimorbidity, which increases with higher age [6], also influencing the needs of home health care. It has been shown that women have a longer life expectancy than men [7], and the longer one lives the higher the risk of multimorbidity. Studies have shown that older women have higher age-adjusted level of multimorbidity [8] as well as more home health care visits and higher medicine consumption than older men, which partly can be explained by the number of chronic diseases [9].

Sweden has one of the oldest populations in the world [10], and the proportion of individuals over 65 years old is increasing [11]. Most older patients are interested in staying at home as long as possible, which increases the demand for home health care [12]. Home health care in Sweden is conducted by a team including physiotherapists, occupational therapists, nurses and GPs that have responsibility for PHC patients in the same area.

To our knowledge no population-based study of home health care and multimorbidity has been conducted before in Sweden. The aim of the study was to study the proportion of the population above 65 years receiving home health care, taking age, gender and multimorbidity into account.

## Methods

The study population comprised people aged 65 or older (32,130 inhabitants), who lived in Blekinge County in southern Sweden (approximately 150,000 inhabitants). We analysed data from patient electronic medical records registered in Blekinge County Council. Our data included the following variables: age, gender and contacts with home health care staff (nurses, physiotherapists and occupational therapists) during 1 year from PHC, and both ambulatory care and inpatient diagnoses from PHC and secondary health care (SHC). The dependent variable was dichotomised into receiving or not receiving home care.

In our study home health care is the medical and rehabilitative care provided by licensed/skilled healthcare professionals to patients living in their own homes. Thus, it does not include home help like domestic aid and personal care. In Blekinge County home health care was provided by district nurses, physiotherapists, occupational therapists, with or without the participation of GPs employed in different PHC centres within Blekinge County Council. Patients eligible for home health care had to be homebound, meaning that they could not leave home without a considerable effort. The home health care was organised as a separate unit within the Blekinge County Council. The GPs were, however, still directly employed at PHC centres.

Patients’ diagnoses from both PHC and SHC were used in order to analyse their level of multimorbidity. Multimorbidity is usually defined as the coexistence of two or more long-term conditions in an individual. In order to group individuals according to the level of multimorbidity we used the Adjusted Clinical Groups Case-Mix System (ACG) [13]. The ACG is software based on the assumption that clustering of illnesses is associated with certain individuals more than others. The classification is thus based on the presence of illnesses and diagnoses in the individual. In order to classify individuals in one of the morbidity levels data files with age, gender and diagnoses are needed. The over 90 ACGs can be then collapsed by the software into six levels of multimorbidity, known as resource utilization bands (RUB). RUB 0 means the lowest and RUB 5 the highest multimorbidity level (0—no or only invalid diagnoses, 1—healthy users, 2—low, 3—moderate, 4—high, 5—very high users) [13]. ACG has been evaluated before both internationally and in Sweden and was a good tool for using patient’s morbidity in the explanation of the use of primary health care services and as a proxy for health care costs in an elderly population [14, 15].

### Statistical analysis

We used STATA version 13 (Stata Corporation, Texas, USA) for statistical analysis. Multiple logistic regression was performed. The dependent variable was the dichotomous variable of receiving home health care (district nurse, occupational therapist, and physiotherapist). The independent variables were age, gender and multimorbidity level.

In order to analyse the differences between individuals receiving home health care and those who did not, we used Chi squared test. Logistic regression analysis was conducted in order to study how the dependent variable was influenced by the independent variables. A result of p < 0.05 was considered statistically significant.

## Results

We analysed data on 32,130 inhabitants of Blekinge County aged 65 or older in 2011(1 January to 31 December 2011). A total of 4240 (13 %) individuals were excluded from analysis due to change of listing status or death during studied period. We found that 7860 (28 %) of the studied population received home health care. A higher proportion of women, especially those above 80 years, received home health care compared to men (Tables 1 and 2).

**Table 1:** Prevalence of having home health care according to gender, age and multimorbidity level

All included			
Home health care	No (%)	Yes (%)	p
Gender			
Female	10,483 (69)	4801 (31)	<0.001
Male	9547 (76)	3059 (24)	
Total	20,030	7860	
Age			
65–69	6257 (90)	704 (10)	<0.001
70–74	5326 (85)	960 (15)	<0.001
75–79	3858 (74)	1378 (26)	<0.001
Over 80	4589 (49)	4818 (51)	<0.001
Total	20,030	7860	
Resource utilization bands			
RUB 0	1313 (84)	260 (16)	<0.001
RUB 1	3043 (85)	558 (16)	NS
RUB 2	4028 (82)	902 (18)	NS
RUB 3	10,046 (71)	4069 (29)	<0.001
RUB 4	1325 (47)	1471 (53)	<0.001
RUB 5	275 (31)	600 (69)	<0.001
Total	20,030	7860

**Table 2:** Prevalence of having home health care in age group and multimorbidity level according to gender

RUB	Female		Male		p
	No (%)	Yes (%)	No (%)	Yes (%)	
65–69 years old					
RUB 0	274 (93)	20 (7)	284 (94)	18 (6)	0.7
RUB 1	512 (96)	20 (4)	540 (98)	12 (2)	0.1
RUB 2	681 (93)	51 (7)	744 (94)	43 (6)	0.2
RUB 3	1516 (88)	205 (12)	1374 (90)	157 (10)	0.1
RUB 4	134 (71)	55 (29)	143 (68)	67 (32)	0.5
RUB 5	19 (41)	27 (59)	36 (55)	29 (45)	0.1
70–74 years old					
RUB 0	154 (89)	19 (11)	178 (90)	20 (10)	0.8
RUB 1	444 (95)	21 (5)	440 (94)	29 (6)	0.3
RUB 2	601 (91)	62 (9)	518 (90)	55 (10)	0.9
RUB 3	1364 (81)	317 (19)	1273 (87)	195 (13)	<0.001
RUB 4	134 (59)	94 (41)	169 (66)	86 (34)	0.09
RUB 5	17 (32)	36 (68)	34 (57)	26 (43)	0.01
75–79 years old					
RUB 0	84 (73)	31 (27)	105 (89)	13 (11)	0.002
RUB 1	291 (87)	45 (13)	245 (90)	28 (10)	0.2
RUB 2	405 (84)	78 (16)	331 (82)	73 (18)	0.5
RUB 3	1107 (72)	425 (28)	959 (75)	316 (25)	0.08
RUB 4	117 (48)	129 (52)	156 (58)	113 (42)	0.03
RUB 5	24 (32)	52 (68)	34 (31)	75 (69)	0.96
Over 80 years old					
RUB 0	137 (60)	90 (40)	97 (66)	49 (34)	0.2
RUB 1	304 (54)	254 (46)	267 (64)	149 (36)	0.002
RUB 2	446 (54)	385 (46)	302 (66)	155 (34)	<0.001
RUB 3	1435 (46)	1655 (54)	1018 (56)	799 (44)	<0.001
RUB 4	226 (29)	541 (71)	246 (39)	386 (61)	<0.001
RUB 5	57 (23)	189 (77)	54 (25)	166 (75)	0.7

*RUB* resource utilization band

We observed that higher age and higher level of multimorbidity (RUB 3–5) were associated with a significantly higher proportion of patients with home health care for both genders (Table 1). A substantial group (22 %) of all who received home health care were patients with low multimorbidity level (RUB 0–2) (Table 1). Logistic regression analysis showed that men had 26 % lower odds of having home health care (OR = 0.74, 95 % CI 0.69–0.78). The odds of receiving home health care were almost nine times higher in women older than 80 compared to those 65–69 years old (OR = 8.9, 95 % CI 7.9–10.1). The oldest men had almost seven times higher odds of receiving home health care (OR = 6.9, 95 % CI 6.1–7.9). When we adjusted for gender and age there were no differences in odds of receiving home health care among patients with lower levels of multimorbidity (RUB 0–2). However, among patients with higher level of morbidity the odds drastically increased (Table 3).

**Table 3:** Odds ratio of having home health care adjusted for gender, age and resource utilization band

	Included in study		Female		Male	
	OR (CI)	p	OR (CI)	p	OR (CI)	p
Gender						
Female	1					
Male	0.74 (0.69–0.78)	<0.001				
Age						
65–69	1		1		1	
70–74	1.53 (1.38–1.71)	<0.001	1.62 (1.41–1.87)	<0.001	1.44 (1.23–1.68)	<0.001
75–79	2.84 (2.57–3.15)	<0.001	2.87 (2.50–3.29)	<0.001	2.82 (2.43–3.27)	<0.001
Over 80	8.01 (7.32–8.76)	<0.001	8.92 (7.90–10.07)	<0.001	6.92 (6.05–7.92)	<0.001
Resource utilization bands						
RUB 0	1		1		1	
RUB 1	0.83 (0.70–0.98)	<0.03	0.81 (0.65–1.02)	0.07	0.85 (0.65–1.11)	0.23
RUB 2	1.04 (0.88–1.22)	0.65	1.00 (0.81–1.24)	0.98	1.09 (0.85–1.40)	0.5
RUB 3	1.66 (1.44–1.93)	<0.001	1.63 (1.34–1.98)	<0.001	1.72 (1.37–2.16)	<0.001
RUB 4	4.09 (3.48–4.82)	<0.001	4.04 (3.25–5.03)	<0.001	4.21 (3.29–5.39)	<0.001
RUB 5	8.38 (6.80–10.34)	<0.001	7.89 (5.87–10.60)	<0.001	8.91 (6.60–12.04)	<0.001

*OR* odds ratio

## Discussion

Our study showed that there are significant differences in odds of receiving home health care, depending on age, gender and multimorbidity level. Being a woman was associated with significantly higher odds of receiving home health care, especially if aged above 80 years. Increasing multimorbidity level increased the odds of receiving home health care to a similar degree in both genders.

Interestingly, we found that a number of patients receiving home health care had a low level of multimorbidity or no morbidity at all. Most of them were older people, and there were a higher number of women in this group.

Our study included all individuals receiving home health care in the county during 1 year. The strength of our study is that we could analyse multimorbidity of all individuals aged 65 and above on an individual level. It enabled us to discover that a large number of individuals without morbidity or with low morbidity used home health care services. The weakness of our study is that in order to see the effects of the PHC centres we had to exclude about 12 % of individuals over 65 years who changed PHC centre or died (most of them) during the period under study. Another weakness is that ACG Case-mix is dependent on diagnoses. Although we have used all the patients’ PHC and SHC diagnoses, if the diagnosis made is not correct, the multimorbidity level assigned might not be valid. In this case home health care might be based on morbidity, even if we did not find any morbidity for this patient in our data set. We did not validate the diagnosis. However, making a diagnosis during the consultations is mandatory, but how complete it is may vary between physicians. An earlier study from Sweden has, however, shown that 75 % of inhabitants had a diagnosis in PHC, and about 90 % of consultations resulted in a diagnosis [16]. In Blekinge County ACG was not used for reimbursement at the time of study, but in other counties a clear increase in diagnosis prevalence, especially at the physician level, was observed when ACG was introduced as a part of reimbursement system [17]. Other earlier studies from different countries have shown that ACG is a good instrument for measuring multimorbidity [13, 18, 19]. However, ACG based multimorbidity level is limited only to diagnosis, so other factors like functional ability level are not included. It is a limitation of our study, as some of the patients with lower functional ability can have more home health care, even if they do not have diagnoses. On the other hand, functional ability is to a certain extent associated with morbidity and should be seen in higher RUB.

Home health care is an integral part of PHC, and it has significance for older patients who cannot come to the PHC centre. In a study from Stockholm patients who had regular home health care provided by district nurses did not need to consult the GPs as often as other patients [5]. Home health care is also used in order to prevent deterioration of their health. A study in Germany showed for example that preventive home visits significantly decreased the number of patient falls [20]. With increasing age the risk of injury connected with falls increases, and a high percentage (more than 40 %) of falls result in hospitalisation [21]. The problem for the elderly with multimorbidity is hospitalisation, but also increased need of institutionalisation.

Our study shows that higher age and higher multimorbidity level were associated with higher odds of receiving home health care both for men and women. However, a higher proportion of elderly women than elderly men received home health care, although they had same multimorbidity level. Earlier studies have shown that older men more often than women identify their spouses as caregivers [22]. This can partly explain why older women more often receive home health care. As women live longer than men, they often take care of their spouses and can help them with contacts to PHC centres, so home health care is unnecessary. The situation for the elderly women might be different when they need health care, as they more often do not have help from a spouse. Elderly women are more often hospitalised, have longer stays at hospitals and have higher rates of most chronic diseases than men [22]. Other studies from Blekinge County showed that multimorbidity level is positively correlated with number of consultations in PHC [23], and that women and individuals with higher multimorbidity level were more often actively listed at PHC [23, 24]. In our study we found that even home health care utilisation is associated with multimorbidity level and gender, especially in older women.

The fact that women use more health care, especially PHC, has previously been reported in other studies [25, 26]. Women also more often feel that their health is poor [27]. We found that women have a higher level of multimorbidity than men, especially the older ones. It is well-known that with increasing age the utilisation of healthcare increases, but the increase is higher for women [28]. Older women have more doctor visits to PHC and generally more visits to PHC, but older men have more visits to specialists [28]. Our results show that older women have higher odds of receiving home health care than older men, even after adjusting for age. Our study has shown, however, that many individuals receiving home health care have no or very low level of multimorbidity. We have not found any direct answer to this in our study. One option may be that there are factors other than diseases, like higher age, perceived subjective health, functional ability level or isolation, which require the necessity of home health care. Further studies are needed in order to identify these factors, in order to be able to provide the whole picture of home health care use.

## Conclusions

In a society with an increasing number of older people living alone, the question of to whom and to what extent home health care should be provided is an important challenge for policy makers. Our results show that there are differences in the use of home health care, depending on gender, age and multimorbidity, but also that home health care is provided to individuals with low or no morbidity. Health needs of an increasing number of older and multimorbid individuals will generate increase in costs of home health care, which should be recognised by policy makers and healthcare managers. Further studies could further explain why the differences appear and what factors influence home health care use by individuals with low morbidity.

## Abbreviations

ACG: adjusted clinical groups
GP: general practitioner
PHC: primary health care
RUB: resource utilization band
SHC: secondary health care
